# Diagnosis of Thyroid Nodules in Children and Adolescents with Subclinical Hypothyroidism and Their Outcomes after Early Thyroxine Treatment—A Longitudinal Study

**DOI:** 10.3390/diagnostics14141528

**Published:** 2024-07-15

**Authors:** Eirini Kostopoulou, Eleana Georgia Koliofoti, Diamantina X. Spilioti, Konstantinos Miliordos, Spyros Skiadopoulos, Andrea Paola Rojas Gil, Sotirios Fouzas, Xenophon Sinopidis, Bessie E. Spiliotis

**Affiliations:** 1 Division of Pediatric Endocrinology and Diabetes, Department of Pediatrics, School of Medicine, University of Patras, 26504 Patras, Greece; irekost@upatras.gr (E.K.); eleana2485@yahoo.com (E.G.K.); diamantinaspilioti@gmail.com (D.X.S.); apaola71@yahoo.com.mx (A.P.R.G.); spilioti@upatras.gr (B.E.S.); 2 Department of Pediatrics, School of Medicine, University of Patras, 26504 Patras, Greece; mhlcon95@gmail.com (K.M.); sfouzas@upatras.gr (S.F.); 3 Department of Medical Physics, School of Medicine, University of Patras, 26504 Patras, Greece; sskiado@gmail.com; 4 Laboratory of Basic Health Sciences, Department of Nursing, Faculty of Health Sciences, University of Peloponnese, 22100 Tripoli, Greece; 5 Department of Pediatric Surgery, School of Medicine, University of Patras, 26504 Patras, Greece

**Keywords:** thyroid nodule, subclinical hypothyroidism, goiter, children, adolescents, thyroid stimulating hormone (TSH)

## Abstract

Pediatric thyroid nodules (TNs) present a higher malignancy rate compared to adults. We sought to diagnose the frequency and characteristics of TNs in children and adolescents with subclinical hypothyroidism (SH) and their outcomes after levothyroxine (LT4) therapy. A total of 256 children with TNs and SH were followed every semester from 2006 to 2018. All patients were treated with LT4. Clinical and radiologic findings, such as the size and texture of the nodules, were documented. Analysis included one-way ANOVA, Kruskal–Wallis, Chi-square, and Fisher’s exact tests. After initial LT4 therapy, TNs disappeared in 85.5% and did not reappear throughout follow-up. In 14.5%, TNs remained the same or increased in size, but they decreased after subsequent LT4 administration with an increased dose. Thyroid disease family history (FHTD) was documented in 77.0%. In total, 64.5% developed a goiter, 46.0% exhibited thyroid heterogeneity on ultrasound, 23.4% had positive Anti-Tg, and 25.4% had positive anti-TPO autoantibodies. Our findings support the possible premise that early pharmacologic intervention with LT4 may be beneficial in children and adolescents with TNs and SH. The increased frequency of FHTD, goiter, thyroid heterogeneity, and Hashimoto in our patients emphasizes that thyroid ultrasounds may be warranted in children and adolescents with these characteristics in order to rule out the presence of TNs.

## 1. Introduction

Thyroid nodules (TNs) are rare in children, with an estimated prevalence of 0.05–1.8% and a range of 0.2–5.1% in ultrasonography [1,2]. Risk factors for the development of TNs include female gender, iodine deficiency, head and neck irradiation, natural goitrogens, and a family history of thyroid diseases [3]. Hormonal growth factors (TSH, IGF-1, EGF, and FGF) may also play a role in their development and proliferation [4].

Thyroid nodules often develop in the setting of Hashimoto’s thyroiditis [5,6,7]. Autoantibody positivity has been reported in 41% of children with a TN and in 1.8–9.6% of children with malignant TNs [8,9].

Despite the low prevalence of TNs in the pediatric population, the rate of malignancy is high, reaching 20–25%, as opposed to 5–10% in adults [10,11,12,13]. Children present more frequently at diagnosis with advanced disease, multifocal disease, lymph node involvement, and distant metastases compared to adults [14]. Hard palpable nodules or firm lymph node enlargement warrant prompt investigation, particularly in the context of present risk factors, such as a history of radiotherapy and a family history of thyroid cancer [15]. It has been suggested that chronic TSH stimulation may play a key role in thyroid cancer pathogenesis. Even modest TSH suppression after LT4 treatment improved the survival of patients with stage II thyroid cancer [16]. Furthermore, a meta-analysis of 28 studies, which included 42,032 patients, showed an increased risk of thyroid cancer in patients with TNs and TSH concentrations higher than 2.5 mU/L [17]. Taking into consideration the results of these studies, it has been recommended by some authors that children who have a goiter or TNs with mild subclinical hypothyroidism should be treated with LT4 [18].

Thyroid ultrasound is the first-line imaging technique for the radiologic evaluation of TNs due to its accuracy, ease of applicability, and its noninvasive character. Although physical examination is quite effective in the detection of TNs localized in the anterior surface or isthmus, ultrasound is more accurate in detecting nodules, particularly in the upper pole of the thyroid [15]. Ultrasound can also distinguish non-nodular diagnoses, such as ectopic thymus and abscesses [14]. It provides information about the size and composition of the TN (cystic, solid, or mixed), thyroid echogenicity, edge irregularity, location, presence of microcalcifications, blood flow in the nodule, and the presence of cervical lymph node alterations. All the above features are important for distinguishing benign from malignant TNs [13,19,20,21]. 

Nevertheless, the thyroid ultrasound alone cannot always definitively distinguish benign from malignant nodules. Thus, systematic monitoring and further evaluation are always needed. In the case of TN diameters over 1 cm and with suspicious sonographic features, thyroid fine-needle aspiration biopsy (FNAB) is recommended [22]. Based on the imaging features and the FNAB findings, a standardized, category-based reporting system for thyroid FNAB specimens has been established, known as The Bethesda System for Reporting Thyroid Cytopathology (TBSRTC) [23,24].

The objective of the present longitudinal study was to document the clinical, radiological, and epidemiological characteristics of children and adolescents from western and southern Greece with thyroid nodules and subclinical hypothyroidism (SH) over a 12-year follow-up period and the outcome of the nodules after LT4 therapy.

## 2. Materials and Methods

This is a longitudinal study of 256 children and adolescents from western and southern Greece with TNs and SH, aged 6 to 18 years, who were recruited from the Outpatient Pediatric Endocrinology Clinic (OPEC) of the University Hospital of Patras, Greece. The patients were followed for up to 12 years (2006–2018). In this study, we defined SH using the cutoff value of >3 mU/L for TSH concentrations, in the presence of FT4 concentrations within the normal range. We based this on the literature where an upper limit of 2.5–3.0 mU/L has been proposed for TSH as being more realistic, since a median TSH concentration in healthy individuals varies between 1.4 and 1.8 mU/L in adults and peaks at approximately 1.5 in children and adolescents [25,26,27,28]. The patients with SH and TNs were part of our larger SH study cohort of 818 patients with clinical symptoms and features such as growth failure, speech delay, psychomotor delay, premature adrenarche, or precocious puberty that were followed-up for a 16-year period (2002–2018).

We studied the clinical characteristics; past, present, and family history of thyroid disease and thyroid cancer; and the biochemical and ultrasonographic thyroid characteristics of the patients. In all the patients, the serum calcitonin concentrations were normal. The TNs were diagnosed during routine check-up in 34% (87/256) of the patients and after an endocrine consultation due to the presence of a goiter in 21% (54/256). The rest of the patients with TNs were diagnosed after referral to the OPEC by their pediatricians due to growth failure, premature adrenarche, or precocious puberty.

All the patients with TNs and SH were treated with an initial dose of 1 μg/kg of LT4. Patients with alternative thyroid diagnoses, such as ectopic thymus or an abscess, were excluded from the cohort.

All the participants were diagnosed with at least one TN via physical examination and/or thyroid ultrasonography. Ultrasonography was performed at the diagnosis of SH and every 6–12 months accordingly. It included color Doppler sonography and elastography.

Thyroid characteristics (thyroid volume and morphology; location, size, and morphology of thyroid nodules; thyroid echogenicity; edge regularity; blood flow in the nodule; and the presence of cervical lymph nodes and their characteristics) were assessed by experienced ultrasonographers of the hospital or identified radiology centers using high-resolution sonography. The length, width, and depth of each thyroid lobe were measured on transverse and longitudinal scans. The thyroid volume was defined as the sum of the volumes of both lobes, without including the isthmus [29]. From 2010, the Thyroid Imaging Reporting and Data System (TIRADS) was also used to evaluate and follow-up all the children with thyroid nodules [30,31].

When indicated, ultrasound-guided fine-needle aspiration biopsy (FNAB) was performed by an experienced radiologist. The FNAB was assessed by an experienced pathologist using The Bethesda System for Reporting Thyroid Cytopathology [22]. 

The data were analyzed with the statistical package SPSS version 24.0 (SPSS, Chicago, IL, USA). The normality of quantitative data was determined with the Shapiro–Wilk test. Descriptive data are expressed as mean ± standard deviation (SD) for quantitative variables, and as numbers with percentages for the categorical variables. Comparisons for the parametric variables were analyzed with one-way ANOVA and the non-parametric with the Kruskal–Wallis test. Spearman’s rho correlation analysis was conducted. Differences between the categorical variables were analyzed with Chi-square and Fisher’s exact test, as appropriate. Significance was evaluated at *p* < 0.05.

## 3. Results

The frequency of TNs in our total study population of 818 patients with SH was 31% (256/818). The majority of patients (85%) had cystic TNs, whereas the remaining 15% had mixed thyroid nodules with cystic and solid components.

The 256 patients with SH and TNs had mean TSH concentrations of 4.26 ± 1.17 mU/L and mean FT4 concentrations of 15.18 ± 0.80 pmol/L. The mean age of the study participants was 9.55 ± 3.46 years (mean ± SD) at the time that SH and TNs were diagnosed.

### 3.1. Thyroid Nodules and Family History 

A family history of thyroid disease (hypothyroidism, Hashimoto thyroiditis, and/or benign TNs) was documented in 197 (77.0%) of the patients with thyroid nodules. Also, twenty patients (7.8%) with a solitary TN had a family history of papillary thyroid cancer in their mother or grandmother (Table 1).

### 3.2. Gender and Pubertal Stage

Of the 256 patients with TNs, 162 (63.3%) were females and 94 (36.7%) were males. Of the 109 patients with one TN, no statistically significant difference was found between gender and pubertal stage (*p* = 0.149). Of the 147 children with more than one TN, there were significantly more pubertal females (*p* = 0.020) (Table 2).

### 3.3. Thyroid Nodules and BMI

Of the 256 patients with TNs, 151 patients (59.0%) were lean (BMI < 85%), 49 patients (19.1%) were overweight (BMI: 85–95%), and 56 patients (21.9%) had obesity (BMI > 95%). 

### 3.4. Thyroid Nodules and Goiter

Sixty-four percent (165/256) of the patients developed a goiter. Among the children with one TN, sixty-one patients (56%) developed a goiter, of which thirty-six patients (59%) were females (*p* = 0.943). Among the children with more than one TN, a goiter was diagnosed in 104 patients (70.7%), of which 68 patients (46.3%) were females (*p* = 0.608). 

### 3.5. Anti-Thyroglobulin (Anti-TG) Antibodies and Anti-TPO Antibodies

In total, 60 patients (23.4%) developed positive anti-TG antibodies and 65 (25.4%) developed positive anti-TPO antibodies. Pubertal patients had significantly higher rates of positive anti-Tg antibodies (*p* = 0.006) and positive anti-TPO antibodies (*p* = 0.001).

### 3.6. Thyroid Heterogeneity

Of the 256 patients, 114 (44.5%) exhibited thyroid heterogeneity on ultrasound. Of the 109 children with one TN, 32 (29.4%) had heterogeneity, 24 of which were female (*p* = 0.026). Of the 147 patients with more than one TN, 82 (55.8%) had thyroid heterogeneity on thyroid ultrasound. Thyroid heterogeneity was present in 57 females (*p* = 0.411) (Table 3).

### 3.7. Number of Nodules 

A total of 43% of the patients had a solitary TN and 57% had more than one. Pubertal and prepubertal patients were almost equally distributed amongst the patients with one TN (54 (49.5%) prepubertal and 55 (50.5%) pubertal), and amongst the patients with more than one TN (70 (47.6%) prepubertal and 77 (52.4%) pubertal).

### 3.8. Size and Texture of Thyroid Nodules

The initial sizes of the TNs on ultrasound in the children and adolescents are shown in Table 4. In total, 85 (78%) of the 109 patients with a solitary TN and 119 (81%) of the 147 patients with more than one TN had an initial thyroid nodule size of ≤5 mm. The majority (77—70.6%) of the 109 children with a solitary TN, had a cystic nodule, whereas the remaining 32 (29.4%) had a cystic and solid (mixed) TN. Also, the majority (138—93.9%) of the 147 children with more than one TN, had cystic nodules, and the remaining nine (6.1%) had mixed TNs.

### 3.9. Thyroid Nodule Outcomes after Initial LT4 Treatment

After the initial LT4 therapy in the patients with both solitary TNs and multiple TNs, the TNs disappeared in 85.5% (219/256) (*p* < 0.01), whereas in 14.5% (37/256) they increased in size or remained unchanged. In the patients in whom the TNs disappeared after the initial LT4 therapy, the TSH concentrations were decreased to the lower limits of normal of the TSH assay, i.e., mean ± SD: 1.12 ± 0.57 mU/L, and the FT4 concentrations were kept within normal limits, while the nodules did not reappear over the entire follow-up period up to the age of 18 years (Table 5).

More specifically, the TNs after the initial LT4 therapy disappeared in 86.2% (94/109) of patients with a solitary TN (*p* < 0.01), of which 77 patients had a cystic TN and 32 children had mixed TNs. In four (3.7%) patients (*p* < 0.01), the size of the TN remained unchanged, being cystic in two patients and a mixed nodule in the other two. In 11 (10.1%) of the 109 patients (*p* < 0.01), the TN increased in size. The TN was cystic in nine and mixed in two of these patients (Table 5).

Similarly, the TN after the initial LT4 therapy in the patients with multiple TNs disappeared in 125 (85%) of the 147 patients (*p* < 0.01), of which they were cystic in 117 patients and mixed in 8. In 3.4% (5/147) of patients (*p* < 0.01), the size of the TN remained unchanged, with all of these TNs being cystic, while in 11.6% (17/147) of patients, the size increased—being cystic in 16 and mixed in 1 patient (Table 5).

In the four patients with a solitary TN that remained the same size after the initial LT4 therapy, the diameters of the TN ranged between 3.5 and 4.0 mm. In the five patients with more than one TN that remained the same, the diameter of the TN ranged from 2.6 to 5.5 mm. 

Of the 11 patients with a solitary TN that increased in size, in 10 patients, the TN diameter ranged from 5.5 to 7.0 mm and in 1 patient with a mixed TN, it increased to 7.7 mm. Of the 17 patients with more than one TN that increased in size, 14 patients had multiple cystic TNs with a range in size of 4.0–8.0 mm. The remaining three patients had mixed TNs with a size range of 6.6–8.0 mm. 

The correlation between the number of TNs and the outcome of their size showed no statistical significance in patients with one or more TN (*p* > 0.05). In all the patients with TNs that remained the same or increased in size, there were no radiological signs consistent with malignancy. On the periodical six-month follow-up after 2010, ultrasonography showed that the majority of patients (93%) had TIRADS scores of TR1, whereas the remaining 7% had TIRADS scores of TR2.

In nine patients (four with a solitary TN and five with multiple TNs, of whom the TN size remained unchanged after initial LT4 therapy), and in 28 patients (11 with a solitary TN and 17 with multiple TN) where the TN size increased, we decided to increase the LT4 dose. After two years of the increased LT4 administration, when the TSH concentrations were mildly suppressed and FT4 was normal, we had the following results: (a) in the 9 patients who previously had unchanged TN size, the TN decreased in size to 1.5–2.0 mm; (b) of the 28 patients where the TN had previously increased in size, in 27 patients the TN decreased in size to 2.5–4.0 mm, but (c) in one adolescent female with a solitary mixed TN and a family history of papillary thyroid cancer, the size of the TN increased to 14 mm (1.4 cm). 

In addition, 31.7% (13/41) of the patients with mixed solid-cystic nodules had obesity. Among the patients with obesity, 23.2% (13/56), and among those with normal weight, 14% (28/200), had mixed solid-cystic nodules. In addition, a higher incidence of nodule growth was observed in patients with obesity and mixed solid-cystic nodules {23% (3/13)} compared to typical-weight patients and mixed solid-cystic nodules {7.1% (2/28)}. 

### 3.10. Thyroid Nodules and Thyroid Cancer

None of the patients with TNs in the present study showed any clinical or radiological signs suspicious for thyroid cancer, except for one adolescent female with a solitary mixed solid-cystic TN and a family history of papillary thyroid cancer in the maternal grandmother, where, despite LT4 therapy, the size of the TN increased from 7.7 mm to 14 mm (1.4 cm) in two years. There was no lymphadenopathy. In this patient, ultrasonography of the TN showed a TIRADS score of TR2 but because of the positive family history of papillary thyroid cancer, it was decided that an FNAB of the TN should be performed. The FNAB cytology was normal, showing only colloid tissue. A decision was then made to perform a total thyroidectomy because of the aggressive increase in size of the TN. The cytology of the tissue from the thyroidectomy showed that the nodule had a macrofollicular architecture with focal pseudopapillary sites and follicular epithelia with sub-round nuclei without atypia consistent with follicular nodular disease with lymphocytic thyroiditis lesions without any evidence of malignancy.

Furthermore, an adolescent male patient, without a family history of papillary thyroid cancer, presented at his first clinic visit with a TSH concentration of 6.89 mU/L, a normal FT4, and a thyroid ultrasound that showed microcalcifications in a solitary 9 mm solid TN with an irregular border. Subsequent thyroidectomy showed a papillary thyroid carcinoma with a BRAFV600E mutation.

## 4. Discussion

Thyroid nodules in the pediatric population represent a serious clinical challenge. In a study of children with a TN without thyroid autoimmunity or a history of radiotherapy, 16% of the patients were diagnosed with thyroid carcinoma (papillary: 73.7%, follicular: 15.8%, and medullary: 10.5%) [9].

For many years, the levels of TSH have been recognized as a potential predictor of thyroid cancer in adults and children. TSH concentrations above 2.1 mU/L have been associated with a higher probability for malignancy in adults [28,32,33,34]. TSH concentrations in children with a mean value above 2.86 mU/L were associated with malignancy probability as well [28,35]. An additional explanation of the association between increased TSH concentrations and cancer development may be that cancer disturbs the thyroid function, resulting in TSH elevation.

It has been reported that the risk for malignant TNs in children is 2.5-fold higher in the presence of a family history of benign thyroid disease and 4-fold higher in the presence of a family history of thyroid cancer [36]. A solitary TN in pediatric patients presents a three to five times greater risk of malignancy, with worse outcome for patients younger than 10 years [37]. It has been recommended that solid and mixed TNs should have a low threshold for serial ultrasound imaging and FNAB or surgical removal in the scenario of developing suspicious features [7,38,39].

The most important finding of our study may be that, for the first time to our knowledge, our results suggest that in the majority of children and adolescents with TNs and SH, with TSH concentrations > 3 mU/L and normal FT4 concentrations, early LT4 treatment may cause the disappearance of the TNs or significantly decrease their size, when TSH concentrations are decreased to the lower limits of normal, while keeping the FT4 concentrations normal.

It is also of note that in our study, only one patient had radiological signs suspicious for thyroid cancer, whereas the rest of the patients did not have any clinical or radiological signs of thyroid cancer for all the monitoring years, even though 20 families had a family history of papillary thyroid cancer and 42.6% of the children had a solitary TN, of which 29.3% were mixed thyroid nodules. A possible explanation for this may be that the early LT4 treatment decreased the TSH concentrations at an early stage of TN development, thus reducing the time period that the thyroid and the nodule were driven by higher TSH concentrations. Only one adolescent female patient with a solitary mixed TN and a family history of papillary thyroid cancer had a thyroid nodule that continued to increase in size (>1 cm) during the follow-up period despite a mildly suppressed TSH concentration after LT4 therapy. Nevertheless, thyroidectomy in this patient did not show any atypia of the thyroid or nodule.

Other interesting findings of our study are that 77% of the TN patients had a positive family history of thyroid disease, primarily Hashimoto’s thyroiditis, and 64.5% of the patients had a goiter. Thyroid heterogeneity on ultrasound was seen in 44.5% of our TN population.

Also, the rate of autoimmune thyroiditis was high in our patients with TNs. Specifically, 23.4% of the patients had positive Anti-Tg and 25.4% had positive anti-TPO autoantibodies. This was more evident in patients with a goiter (14.6%, as opposed to 11.6% of the patients without a goiter). These results are higher than those reported for the general population, where 10–13% of most populations and 10% of the Greek pediatric population have been reported to have positive anti-TG or anti-TPO antibodies [24,40,41,42].

Furthermore, a female and pubertal predominance was identified in our TN patients. This has been previously reported for children and, particularly, adolescents with a 3-fold female-to-male ratio [2,7].

One of the limitations of this study is the fact that the thyroid ultrasounds were performed by different ultrasonographers, all of whom, however, were experienced in thyroid ultrasonography in the pediatric population. Another limitation of this study is that our definition of subclinical hypothyroidism is not universally accepted, since the establishment of the upper limit of normal TSH in different age groups poses a challenge.

## 5. Conclusions

In conclusion, thyroid nodules in children and adolescents represent an entity that is frequently discovered in clinical practice during thyroid palpation or imaging, and warrants a careful diagnostic and follow-up approach due to its malignant potential. The present study provides further insight into the association between the presence of thyroid nodules and thyroid comorbidities (SH, goiter, thyroid autoimmunity, and heterogeneous echogenicity), nodular characteristics, and epidemiological parameters in a cohort of children and adolescents with benign thyroid nodules, extending the existing knowledge in this field. Also, the increased frequency of FHTD, goiter, thyroid heterogeneity, and Hashimoto in our patients emphasizes that thyroid ultrasounds may be justified in children and adolescents with these characteristics to rule out the presence of thyroid nodules.

The findings of our study also support the hypothesis that early pharmacologic intervention with LT4 in children with thyroid nodules and subclinical hypothyroidism may possibly be beneficial. Of course, more studies need to be conducted incorporating larger populations of children and adolescents with thyroid nodules to confirm our findings.

## Figures and Tables

**Table 1:** Number of prepubertal and pubertal patients with one or more thyroid nodules (TNs) in relation to family history.

Age Status	Family History						
	One TN			More Than One TN			
	Negative	Positive	Total	Negative	Positive	Total	Total
Prepubertal	12	42	54	17	53	70	124
Pubertal	15	40	55	15	62	77	132
Total	27	82	109	32	115	147	256
	*p* = 0.541			*p* = 0.481			

**Table 2:** Number of prepubertal and pubertal patients with one or more thyroid nodules (TNs) in relation to gender and pubertal stage.

Gender	One TN			More Than One TN			
	Prepubertal	Pubertal	Total	Prepubertal	Pubertal	Total	Total
Female	28	36	64	40	58	98	162
Male	26	19	45	30	19	49	94
Total	54	55	109	70	77	147	256
	*p* = 0.149			*p* = 0.020			

**Table 3:** Number of patients with one or more TN in relation to gender and thyroid heterogeneity.

Gender	Heterogeneity						
	One TN			More Than One TN			
	Negative	Positive	Total	Negative	Positive	Total	Total
Female	40	24	64	41	57	98	162
Male	37	8	45	24	25	49	94
Total	77	32	109	65	82	147	256
	*p* = 0.026			*p* = 0.411			

**Table 4:** Initial ultrasound size of thyroid nodules before LT4 therapy.

Nodule Size (mm)	With One Nodule		With More Than One Nodule	
	*n*	%	*n*	%
2.0–2.5	26	23.9	57	38.8
2.6–3.0	18	16.5	19	12.9
3.1–3.5	9	8.3	11	7.5
3.6–4.0	14	12.8	11	7.5
4.1–4.5	2	1.8	5	3.4
4.6–5.0	16	14.7	16	10.9
5.1–5.4	4	3.7	1	0.7
5.5–6.0	6	5.5	4	2.7
6.1–6.5	0	0.0	2	1.4
6.6–7.0	5	4.6	7	4.7
7.1–7.5	2	1.8	3	2.0
7.6–8.0	1	0.9	2	1.4
8.1–8.5	0	0.0	0	0.0
8.6–9.0	0	0.0	4	2.7
9.1–9.5	0	0.0	1	0.7
9.6–9.8	6	5.5	4	2.7
Total	109	100.0	147	100.0

**Table 5:** Alteration in thyroid nodule size after initial LT4 therapy.

	Disappearance	Same Size	Increased Size	Total
Solitary thyroid nodule	94	4	11	109
Multiple thyroid nodules	125	5	17	147
Total	219	9	28	256
Cystic thyroid nodules	187	5	23	215
Mixed solid and cystic thyroid nodules	32	4	5	41

## Data Availability

Data are available at request by the authors.
